# The Enterotoxin Production and Antimicrobial Resistance of *Campylobacter* Strains Originating from Slaughter Animals

**DOI:** 10.3390/pathogens11101131

**Published:** 2022-09-30

**Authors:** Beata Wysok, Joanna Wojtacka, Agnieszka Wiszniewska-Łaszczych, Marta Sołtysiuk, Aleksandra Kobuszewska

**Affiliations:** Department of Veterinary Public Health, Faculty of Veterinary Medicine, University of Warmia and Mazury in Olsztyn, Oczapowskiego 14, 10-719 Olsztyn, Poland

**Keywords:** *Campylobacter*, suckling mouse assay, CHO assay, enterotoxicity, antimicrobial resistance

## Abstract

The pathogenicity of animal-origin *Campylobacter* strains, including antimicrobial resistance and enterotoxigenicity, was determined in this study. Overall, 149 *Campylobacter* isolates originating from cattle, swine and poultry were tested. The antimicrobial resistance profiles were examined by the diffusion disk method. The dominant resistance pattern was CIP_TET. The resistance rates for ciprofloxacin among swine, cattle and poultry isolates were 84%, 51% and 66%, respectively; for tetracycline, they were 82%, 57.1% and 76%, respectively. None of the obtained isolates was resistant to all four antimicrobials tested. The ability to produce enterotoxins was assessed by the use of a suckling mouse bioassay, with intestinal fluid accumulation as a positive result, and by CHO assay, with the elongation of cells as a positive result. The ability to produce enterotoxins was significantly higher among cattle isolates (61.2% and 71.4% positive isolates, respectively, in the bioassay and the CHO assay) than among swine (16% and 32% positive isolates, respectively) or poultry isolates (14% and 22% positive isolates, respectively). A strong positive correlation between in vitro and in vivo enterotoxicity tests was demonstrated.

## 1. Introduction

Although *Campylobacter* is considered a leading cause of human gastroenteritis worldwide [1], extragastrointestinal infections, including reactive arthritis, Guillain–Barre Syndrome (GBS), bacteremia, septicemia, septic arthritis, and meningitis may also occur [2]. According to the latest report of the European Food Safety Authority and the European Center for Disease Prevention and Control (EFSA and ECDC) [3], the number of confirmed cases of human campylobacteriosis in 2020 was 120,946. Interestingly, for all zoonoses except trichinellosis and yersiniosis, there was a reduction in notification rates in 2020 compared with 2019; this was influenced by the pandemic and by the withdrawal of the United Kingdom from the European Union. However, the overall campylobacteriosis trend in the period 2016–2020 showed no statistically significant increase or decrease. The overall notification rate in 2020 was 40.3 per 100,000 population, although the rates varied considerably between countries, from 1.1 in Poland to 163.8 in Czechia. This was mainly due to how the data were collected and processed, as the estimation of the incidence of campylobacteriosis in the population is usually based on confirmed cases corrected for several factors, such as the proportion of patients consulting a physician and the number of such patients who submit a stool sample for confirmation of the prevalence of *Campylobacter* cells in feces [4]. Because the infection is usually self-limiting, the true population incidence is estimated to be 8 to 30 times higher than the number of confirmed cases, depending on the country [4]. Animal-origin products are considered to be the main source since campylobacters colonize the mucus layer of the intestinal tract of a wide range of animals, mainly birds and mammals; this is recognized as their reservoir [5,6]. Moreover, the animals that harbor *Campylobacter* in their intestinal tract are generally asymptomatic carriers and can easily contaminate the environment and other animals [7,8,9]. Although poultry and poultry products are considered a major source of *Campylobacter* infection [10,11], swine and cattle are also involved in campylobacteriosis in humans [12,13,14]. Most *Campylobacter* infections in humans occur as sporadic cases, and outbreaks are rare. In 2020, 1319 illnesses out of 120,946 confirmed cases were implicated in 317 food-borne outbreaks [3]. Campylobacteriosis in humans is induced mainly by *Campylobacter jejuni* (about 90% of cases), and the remaining fraction is induced predominantly by *Campylobacter coli*. [4]. Gastrointestinal infections caused by *Campylobacter* are generally characterized by diarrhea [2], but patients may also experience fever, weight loss, and cramps that last, on average, 6 days [15]. Diarrhea may range in severity from loose stools to massive watery or bloody stools [16]. The clinical manifestation of campylobacteriosis is mainly due to toxin production. The toxins, according to their biochemical properties, are divided into enterotoxins and cytotoxins. It has been suggested that enterotoxin production results in the watery type of diarrhea, as opposed to inflammatory bloody diarrhea due to cytotoxin production [17]. Enterotoxins elevate the intracellular level of cyclic adenosine monophospate, and the subsequent ion flux changes cause the excess secretion of fluid, which results in watery diarrhea. In contrast, cytotoxic activity appears to be distinct from enterotoxin activity, as cytotoxins are associated with the destruction of target cells causing mucosal inflammation and hemorrhagic reactions [18,19]. However, although several *Campylobacter* cytotoxins have been identified, only cytolethal distending toxin (CDT) has been well characterized, and it was found that CDT activity is encoded by three closely linked or slightly overlapping genes termed *cdtA*, *cdtB* and *cdtC* [20,21]. Simultaneously, there is a lack of identification of the genetic factors associated with enterotoxin production. The capacity of bacteria to produce enterotoxins can be determined by in vitro assay by observing the elongation of CHO (Chinese hamster ovary) cells and the rounding of Y-1 epithelial cells, which are commonly used in toxicology research [17], or by animal models. According to Pore et al. [22], in vivo assays are extremely imperative because they permit researchers to illuminate the complex biological mechanisms that trigger cellular processes and molecular functions in infection and diseases. Moreover, Lipps [23] underlined that there is no in vitro test that can recognize all types of toxins collectively, whereas animal bioassays recognize toxicity from all types of bacterial toxins. Several animal models have been proposed to enhance the virulence of *Campylobacter* spp. [24]. The toxigenic capacity may be determined by the rat intestinal loops model that results in intestinal accumulation due to the altering of the electrolyte exchange [25,26]. Moreover, some authors suggested that enterotoxin produced by *Campylobacter* spp. may be part of the cholera-*Escherichia coli* family of immunologically related heat-labile adenylate cyclase-stimulating enterotoxins [27,28]. As the suckling mouse assay is widely used for the detection of *E. coli* enterotoxin, this model seems to also be relevant for detecting enterotoxins produced by *Campylobacter*.

In relation to the risk factors affecting the course of *Campylobacter* infection, the spread of antibiotic-resistant bacteria is also a major public health concern. In recent years, the increasing antimicrobial resistance of *Campylobacter* strains originating from food of animal origin has been noted [29,30]. However, many cases of campylobacteriosis are self-limiting and require only supportive therapy limited to fluid and electrolyte replacement therapy. Antibiotics are given, particularly when the symptoms are severe or prolongated [31]. The global spread of antibiotic-resistant *Campylobacter* strains is a continuous process due to the regular use of antibiotics in animal husbandry, and this is a problem of public health concern [32]. Macrolides and fluoroquinolones are the most common antimicrobial agents used in the treatment of *Campylobacter* infections [33], while tetracyclines have been suggested as an alternative choice in the treatment of clinical campylobacteriosis [34]. The problem with the rapidly developing resistance to antimicrobial agents, particularly to fluoroquinolones, has been observed in different geographical regions [35,36]. Therefore, it is essential to evaluate the antimicrobial susceptibility of *Campylobacter* strains, especially those originating from food animals that are known to have a high carriage of these bacteria [37].

The aim of this study was to examine the frequency of enterotoxin production among *Campylobacter* isolates originating from slaughter animals by undertaking both in vitro and in vivo tests. Moreover, susceptibility testing was performed to evaluate the sensitivity of the obtained isolates to four classes of antimicrobials.

## 2. Material and Methods

### 2.1. Bacterial Strains

A total of 149 *Campylobacter* isolates, including 50 isolates from poultry, 49 from cattle and 50 from swine, were tested. The isolates were recovered from swabs taken from poultry, swine and cattle carcasses in slaughterhouses before the chilling stage. All samples were collected from January 2012 to February 2013 from the same district in Poland. The swabs were transferred to 0.1% sterile peptone water, and aliquots (2 mL) from each sample were transferred to 18 mL of Bolton broth (Oxoid, Basingstoke, UK) and incubated microaerobically (5% O_2_, 10% CO_2_ and 85% N_2_) at 42 °C for 24 h. After the enrichment, the samples were subcultured on the surface of charcoal cefoperazone deoxycholate modified agar (mCCDA, Oxoid, Basingstoke, UK) and agar Karmali (Oxoid, Basingstoke, UK). Subsequently, the bacterial growths on plates were evaluated for morphologically typical *Campylobacter* colonies. Genotypic confirmation of isolates was performed using a multiplex PCR assay with species-specific primers. (Table 1). Amplification was performed in a 50 μL reaction mixture containing 5 μL of the PCR buffer (10—times concentrated), 5 μL of dNTPs (final concentration of 200 µM), 0.5 μL of each primer (final concentration of 0.1 µM), 10 μL MgCl2 (final concentration of 5 mM), 2 μL (2 U) thermostable Taq polymerase (Termo Fisher Scientific, Waltham, MA, USA), 5 μL of template DNA at the final concentration of 120 ng verified by Nano-DropTM Spectrophotometer (Thermo Fisher Scientific, Waltham, MA, USA) and DNase-and RNase-free deionized water. All PCR reactions were carried out using the following conditions: initial denaturation at 94 °C for 5 min followed by 30 cycles of denaturation for 1 min at 95 °C, annealing for 1 min at 58 °C and extension for 1 min at 72 °C. The final elongation step was carried out at 72 °C for 5 min. A positive control consisting of DNA extracted from *C. jejuni* ATCC 33291 and *C. coli* ATCC 43478, as well as a negative PCR control consisting of PCR-grade water were included in each PCR run. The procedure for the sampling and identification of the isolates obtained was performed as described previously [13,38].

### 2.2. Enterotoxin Production Assay

#### 2.2.1. Preparation of Culture Filtrates

*Campylobacter* strains were inoculated into Brucella broth supplemented with 1% (*v*/*v*) IsoVitaleX (Thermo Fisher Scientific, Waltham, MA, USA) and incubated in microaerophilic conditions (5% O_2_, 10% CO_2_ and 85% N_2_) for 48 h at 37 °C in a rotary shaking water bath. Polymyxin B was added to the suspension 10 min before the end of the incubation at a concentration of 0.15% (*w*/*v*). Broth cultures were centrifuged at 1500× *g* for 20 min at 4 °C, and the supernatants were passed through a membrane filter with a pore size of 0.22 µm (Millipore Corp., Burlington, MA, USA). The prepared culture filtrates were used immediately after preparation.

#### 2.2.2. The Influence of Enterotoxin on CHO Cells

CHO cell lines obtained from the American Type Culture Collection (ATCC, Manassas, VA, USA) were used for the detection of enterotoxins, according to the procedure described previously by Belbouri and Mégraud [40] with slight modifications. Freshly trypsinized cells were suspended in flat-bottom 96-well plates at a density of 1 × 10^5^ cells per well in a volume of 200 µL, and 100 µL of prepared bacteria-free culture supernatants was subsequently added to the monolayers. After incubation in a 5% CO_2_ atmosphere at 37 °C for 24 h, the percentage of elongated cells was determined by counting a minimum of 100 cells per well each time. A sample was considered positive if a minimum of 30% of the cells were elongated. Tests were performed in duplicate for each sample. Simultaneously, a positive control (*E. coli* ATCC 43886) and negative control (phosphate buffer saline, PBS) were included in each test.

#### 2.2.3. Suckling Mouse Assay

BALB/c mice (Mossakowski Medical Research Centre of the Polish Academy of Sciences in Warsaw) were bred in the Laboratory Animal Facility of the Department of Pathophysiology, Forensic Veterinary Medicine and Administration, Faculty of Veterinary Medicine, University of Warmia and Mazury, in Olsztyn, Poland. Permission for all experimental procedures was granted by the Local Ethical Commission for Experiments on Animals in Olsztyn, Poland. The assay was performed according to the procedure described previously by Giannela [41] with slight modifications. Prepared culture filtrates were tested in 3–5-day-old mice. Newborn BALBc suckling mice were separated from their mothers immediately before use and randomly divided into groups of three, as each isolate was tested in three mice. Each mouse was inoculated intragastrically by percutaneous injection with 0.1 mL of a previously prepared culture filtrate containing 2 drops of 2% Evans blue per ml. Then, 3 h following inoculation, the mice were killed by an overdose of anesthesia with isoflurane (Aerrane, Baxter S.A., Deerfield, MA, USA). The abdomen was opened, and the entire intestine, except the stomach, was removed with forceps. Animals with no dye in the intestine or dye within the peritoneal cavity at autopsy were discarded. The intestines from each group of three mice were pooled and weighed, and the ratio of intestinal weight to remaining body weight (IW/RBW) was calculated. A ratio greater than 0.083 was considered positive, whereas between 0.075 and 0.082 was a doubtful result and a ratio less than or equal to 0.074 was a negative result.

### 2.3. Antimicrobial Resistance

Antimicrobial resistance was examined by the diffusion disk method according to the protocol of the European Committee on Antimicrobial Susceptibility Testing (EUCAST) for fastidious organisms. The set of antimicrobials was selected in order to reflect their importance both for human and veterinary medicine and was recommended by EUCAST and ECDC (European Centre for Disease Prevention and Control). All *Campylobacter* isolates were suspended into sterile saline (0.85% NaCl) to a turbidity equivalent to a 0.5 McFarland standard. Mueller–Hinton agar plates supplemented with 5% of defibrinated horse blood (Oxoid) and 20mg/L β-NAD (Sigma Aldrich, St. Louis, MO, USA) were inoculated with the prepared suspension. The following antibiotic disks were placed on the surface of dry plates: erythromycin (ERY, 15 µg), gentamicin (G, 10 µg), ciprofloxacin (CIP, 5 µg) and tetracycline (TET, 30 µg). The plates were incubated at 41 ± 1 °C for 24–48 h in a microaerophilic atmosphere. According to the European Union protocol for the harmonized monitoring of antimicrobial resistance in human *Salmonella* and *Campylobacter* isolates, the interpretation of results for the obtained isolates was based on the epidemiological cut-off value (ECOFF) [42].

### 2.4. Statistical Analysis

All analyses were performed using Statistica (StatSoft, version 13.3, Kraków, Poland). A Kruskal–Wallis nonparametric analysis of variance test was used to perform multiple comparisons between the source of the isolates and the ability to produce enterotoxin or antimicrobial resistance. Statistical significance was defined as *p* < 0.05. An estimation of covariance matrices was performed to measure the strength of the associations between the in vitro and in vivo enterotoxicity tests.

## 3. Results

### 3.1. Enterotoxin Production Assay

#### 3.1.1. The Influence of Enterotoxin on CHO Cells

**Figure 1:**
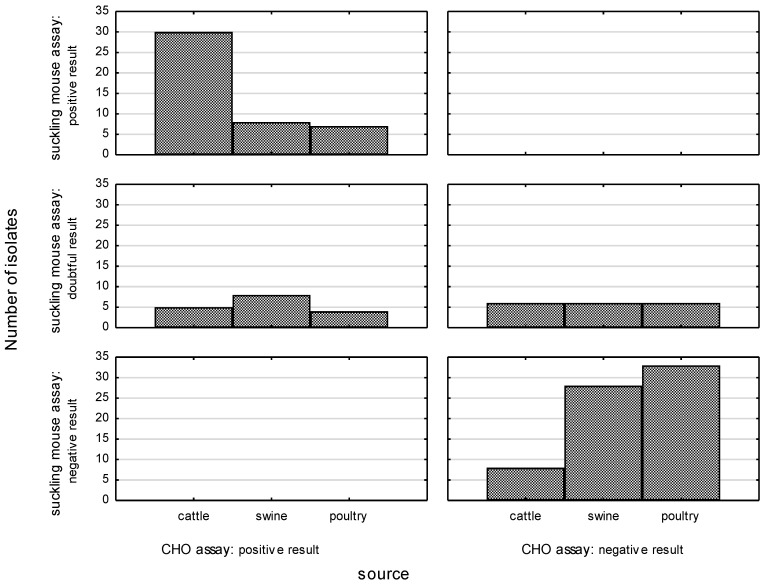
Correlation between the enterotoxic activity of *Campylobacter* strains in CHO and suckling mouse assays.

Of 149 strains tested, a total of 62 (41.6%) revealed enterotoxin activity in the CHO assay. There were significant differences, in terms of exhibiting enterotoxic effects, between isolates recovered from different sources (*p* < 0.05) (Figure 1). The ability to produce enterotoxin was significantly more common among culture filtrates originating from cattle isolates (71.4%) than in filtrates from swine isolates (32%) or poultry isolates (22%).

#### 3.1.2. Suckling Mouse Assay

Of 149 strains tested, a total of 45 (30.2%) were found to be positive for the production of enterotoxin, and the highest rate of enterotoxin activity was noted among bovine-origin isolates (*p* < 0.05) (Figure 1). The ability to cause fluid accumulation in the intestinal tract of suckling mice was confirmed in 30 (61.2%) culture filtrates originating from cattle-origin *C. jejuni* isolates, while 11 (22.4%) isolates yielded doubtful results. The remaining isolates did not show any toxigenic activity. In turn, the majority of *Campylobacter* isolates of swine origin were not able to produce enterotoxin (58% of the isolates); this was true of 50% of *C. jejuni* and 58.7% of *C. coli* isolates, respectively. Overall, 16% of *Campylobacter* isolates (15.2% of *C. coli* and 25% of *C. jejuni*) showed toxigenic activity in the bioassay. For the remaining isolates, doubtful results were reported. Among *Campylobacter* isolates from poultry, enterotoxin production was confirmed in 33.3% of *C. coli* and 12.8% of *C. jejuni* isolates. Doubtful and negative results were noted in 33.3% and 33.3%, respectively, of *C. coli* and in 19.1% and 68.1%, respectively, of *C. jejuni* isolates.

A strong association between in vitro and in vivo enterotoxicity tests was also noted, and the correlation coefficient was calculated as 0.843882 in poultry, 0.825029 in swine and 0.851874 in cattle isolates. All culture filtrates found to be positive in the bioassay were also positive in the CHO assay. A few isolates that yielded doubtful results in the suckling mouse assay were found to have a positive response in cell culture (45.4% in cattle-origin isolates, 61.5% in swine-origin isolates and 40% in poultry-origin isolates).

### 3.2. Antimicrobial Resistance

The highest frequency of resistance was noted for ciprofloxacin (in 84% of swine, 51% of cattle and 66% of poultry isolates) and tetracycline (in 82% of swine, 57.1% of cattle and 76% of poultry isolates). The rates of erythromycin resistance were low in the current study: 14% of swine isolates, 6.1% of cattle isolates and 4% of poultry isolates were resistant to this agent. In total, only one *C. jejuni* isolate from swine was resistant to gentamicin (Table 2). Multidrug resistance, defined as resistance to three or more antibacterial classes, was observed in eight swine isolates (16%), two bovine isolates (4.1%) and two poultry isolates (4%). The dominant resistance pattern was CIP_TET, which was observed in 27 swine isolates (54%), 16 bovine isolates (32.6%) and 25 poultry isolates (50%). None of the obtained isolates was resistant to all four antimicrobials tested. Simultaneously, 4% of swine-origin isolates, 28.6% of bovine-origin isolates and 12% of poultry-origin isolates were sensitive to all tested antibacterial classes.

## 4. Discussion

The burden of campylobacteriosis as a food-borne disease is global; therefore, it requires global efforts in terms of collaboration, funding, awareness and commitment from various governments [43].

Pathogenic bacteria, including bacteria belonging to the genus *Campylobacter*, utilize a number of mechanisms to cause disease in human hosts [44]. Among the factors determining the virulence of *Campylobacter* strains, the ability to colonize and invade the intestinal mucosa plays a key role, although the production of toxins has a significant influence on the disease course. A cytopathic effect correlating with cytotoxin production was confirmed by gene expression analysis in different cell cultures [13,38,45]; however, the production of enterotoxin by *Campylobacter* is still controversial [19]. The dominant perception is that *Campylobacter* enterotoxin is immunologically related to *Vibrio cholere* enterotoxin (CT) or *Escherichia coli* heat-labile enterotoxin (LT) [18]. The previous studies demonstrated that the capacity of *Campylobacter* spp. to produce enterotoxins may be demonstrated by both in vivo and in vitro tests [46]. The current study found a strong correlation between in vitro and in vivo enterotoxicity tests. All culture filtrates positive in the suckling mouse assay also yielded positive results in a cell culture, causing the rounding of CHO cells, while all filtrates without confirmed enterotoxic activity in the bioassay were negative in the CHO assay. The slightly higher ratio of positive results noted for the in vitro assay was due to the fact that some *Campylobacter* isolates that yielded doubtful results in the suckling mouse bioassay were positive in the cell culture assay. Moreover, Ruiz-Palacios et al. [25] observed that filtrates of *Campylobacter* strains obtained from patients with diarrhea promoted fluid accumulation in rat intestinal loops, and positive results were verified by CHO cell assay. The common ability of *Campylobacter* strains to produce enterotoxins was noticed in 75% of isolates from patients with diarrhea in Mexico [25], in 64% of isolates derived from diarrheal diseases of children in Algeria [40] and in 62% of isolates originating from children with *Campylobacter*-induced diarrhea in Costa Rica [47]. Chattopadhyay et al. [48] demonstrated enterotoxigenic activity among *Campylobacter* isolates obtained from both human and animal samples. Simultaneously, those authors noted the approximate amount of fluid accumulation in ligated rat ileal loops that was produced by isolates recovered from fecal samples of chicken handlers with diarrhea, apparently healthy animal handlers, dogs with diarrhea and chickens. However, some authors have been unable to detect enterotoxin production by *Campylobacter* strains [49,50]. According to Akhtar and Huq [51], the detection of toxins depends on precise culture conditions and whether polymyxin B was present or cultures were sonicated. This may explain why there is a lack of confirmation of enterotoxin production by *Campylobacter* strains in some studies recovered from different sources, including a poor correlation for the determination of toxic properties of *Campylobacter* isolates from clinical series with clinical features of the initial illness [52]. The current study is the first study describing enterotoxic activity among *Campylobacter* isolates recovered from slaughter animals. Moreover, this study provides new data on the enterotoxigenicity of *Campylobacter* strains after more than 20 years from previous surveys. Our studies revealed that enterotoxin activity is more common among supernatants derived from cattle-origin isolates (61.2% positive results in the bioassay and 71.4% positive results in the cell culture assay) than among supernatants of swine (16% and 32%) and poultry (14% and 22%) origin. Interestingly, cattle are usually described as a less common source of *Campylobacter* spp. compared to poultry, but these animals, as was shown, may harbor highly pathogenic *Campylobacter* strains. Moreover, we found that enterotoxin production was more frequent in *C. jejuni* than in *C. coli* strains. In total, the production of enterotoxin was detected in 37 of 100 (37%) and 8 of 49 (16%) *C. jejuni* and *C. coli* strains tested in bioassay, respectively, and 47 of 100 (47%) and 15 of 49 (30.6%) strains in cell culture assay, respectively. These findings are in accordance with the results obtained by Belbouri and Megraud [40], who noticed that 74% of *C. jejuni* and 9% of *C. coli* isolates obtained from diarrheic children were positive for enterotoxigenicity. Given the above, the high level of enterotoxigenicity of cattle-origin isolates (71.44 and 61.2% positive strains, depending on the method used) may indicate that *C. jejuni* is a dominant species colonizing the intestines of cattle.

Antimicrobial resistance (AMR) is currently one of the most serious global public health threats [53]. Infections caused by resistant bacteria lead to up to two-fold higher rates of adverse outcomes compared with similar infections caused by susceptible strains [54]. Although antimicrobial resistance has a direct impact on human health, a substantial proportion of total antibiotic use occurs outside the field of human medicine. Antimicrobial use in food-producing animals and in aquaculture for growth promotion and for disease treatment or prevention is probably a major contributor to the overall problem of resistance [55]. Humans are exposed to resistant bacteria through various sources, such as food products or the environment [43]. Tetracyclines and quinolones are antibiotics widely used in veterinary medicine, which results in a high frequency of bacterial resistance to these agents. The extensive development of resistance to these antimicrobial agents in various countries has led to a decrease in their clinical use [56]. In the current study, the highest resistance rates for *Campylobacter* isolates, regardless of the source, were found for ciprofloxacin, ranging from 51% among bovine isolates to 84% among swine isolates; for tetracycline, the rates ranged from 57.1% among bovine isolates to 82% among swine isolates. A similarly high rate of resistance to quinolones and tetracyclines in *Campylobacter* isolated from slaughter animals was reported previously in Poland [30,57], northern Thailand [58] and Switzerland [59]. However, following the European Union summary report, the percentage of resistant *Campylobacter* isolates may vary substantially between E.U. countries [60]. The levels of ciprofloxacin resistance among pig-origin isolates ranged from 16.8% in Estonia to 97.1% in Spain; for tetracycline, it ranged from 0% in Finland to 100% in Spain. For bovine isolates, the highest levels of ciprofloxacin and tetracycline resistance were reported in Croatia (100%) and Italy (91.8%), whereas the lowest rates were reported in the Netherlands (23.3%) and Denmark (6.8%). Due to high rates of resistance to fluoroquinolones and tetracyclines, those agents should not be considered highly effective in the treatment of *Campylobacter* infection.

In the treatment of *Campylobacter* infections, macrolides such as erythromycin or azithromycin are usually recommended. This is largely due to the opinion that the resistance of *Campylobacter* isolates to macrolides has remained stable at a low level. This low level of resistance to erythromycin was commonly noted among *Campylobacter* isolates, regardless of source or geographical area. The erythromycin-resistant strains represented 2% of human-origin strains in Belgium [61], 4.3% of cattle strains in Poland [62], 3.98% of poultry strains in Canada and none of the swine strains in Sweden [63]. In the current study, a similarly low rate of resistance to erythromycin was observed among poultry and cattle isolates (4% and 6.1% resistant isolates, respectively). Although erythromycin resistance was more frequently noted among *Campylobacter* isolates of swine origin (14%), the rate was higher among *C. coli* isolates (25%) than *C. jejuni* isolates (13.0%). It has been suggested that the incidence of macrolide resistance among *Campylobacter* isolates is highly variable with respect to the source of the isolate origin and the frequency and type of antimicrobial agents used for treating animal and human infections in different geographical areas [35]. According to Bollinger et al. [64], the high level of resistance to erythromycin in *C. coli* strains is possibly due to the greater survival capacity of strains with mutations associated with resistance to macrolides when compared to *C. jejuni*. Moreover, in most countries, the prevalence rate of gentamicin-resistant *Campylobacter* is low [65,66,67]. In the current study, only a single isolate of swine origin was resistant to gentamicin. A aminoglycosides are a class of broad-spectrum antibacterial agents used for the treatment of both Gram-positive and Gram-negative organisms. Gentamicin is an important aminoglycoside and is used in human beings for the treatment of severe infections, including systemic infections caused by *Campylobacter* [68]. However, in some studies, the observed rising resistance rate to gentamicin was described as a novel phenomenon [69]. Schiaffino et al. [70] reported the prevalence of gentamicin-resistance as 15.8% among non-*C. jejuni* and 1.0% among *C. jejuni* isolates in a pediatric study in Peru. Moreover, Pan et al. [71] estimated that 28.8% of resistant isolates originated from human diarrheal samples from China. Zhao et al. [69] indicated that the emergence of gentamicin resistance in *Campylobacter* probably resulted from the horizontal transfer of resistance genes from other microorganisms. However, the generally low resistance rates noted in E.U. countries for erythromycin and gentamicin favor these antimicrobials as the first-choice recommendation.

The current study underlined the emerging public health problem due to the increasing number of isolates resistant to more than one antimicrobial agent. The vast majority of isolates (54% of swine isolates, 32.6% of cattle isolates and 50% of poultry isolates) were resistant to both tetracycline and ciprofloxacin. This resistance pattern seems to be prevalent among isolates, regardless of origin, in different geographical regions, e.g., in Italy [72], the U.S.A. [73] and Lithuania [74]. According to Wieczorek et al. [75], the increasing resistance among *Campylobacter* to macrolides, combined with resistance to quinolones, is especially disturbing. Moreover, it was shown that human infections caused by macrolide- or quinolone-resistant *Campylobacter* are associated with an increased risk of adverse events or development of the invasive form of the disease compared to infections with susceptible isolates [76].

In summary, the current study indicates that slaughter animals should be recognized as a source of pathogenic *Campylobacter* strains able to produce enterotoxins. In particular, cattle, often described as a less common source of *Campylobacter* spp., should be recognized as an important source of enterotoxigenic *Campylobacter* strains. Regarding the antibiotic therapy used in the treatment of a severe course of campylobacteriosis, macrolides and aminoglycosides should be recommended.

## Figures and Tables

**Table 1:** PCR primers used in the study.

Target Gene	Sequences (5′–3′)	PCR Product Size (bp)	**References**
*16S rRNA* for *Campylobacter* spp.	F—ATCTAATGGCTTAACCATTAAAC R—GGACGGTAACTAGTTTAGTATT	857	[39]
*mapA* for *C. jejuni*	F—CTATTTTATTTTTGAGTGCTTGTG R—GCTTTATTTGCCATTTGTTTTATTA	589	[39]
*ceuE* for *C. coli*	F—AATTGAAAATTGCTCCAACTATG R—TGATTTTATTATTTGTAGCAGCG	462	[39]

**Table 2:** The resistance profiles among *Campylobacter* isolates originating from slaughter animals.

Resistance Pattern	Swine Isolates		Poultry Isolates		Cattle Isolates
	*C. coli* (*n* = 46)	*C. jejuni* (*n* = 4)	*C. coli* (*n* = 3)	*C. jejuni* (*n* = 47)	*C. jejuni* (*n* = 49)
CIP_E_TET	6	1	−	2	2
CIP_TET_G	−	1	−	−	−
CIP_TET	26	1	1	24	16
CIP_E	−	−	−	−	1
TET	6	−	1	10	10
CIP	7	−	1	5	6
E	−	−	−	−	−

*Abbreviations:* CIP—ciprofloxacin, TET—tetracycline, E—erythromycin, G—gentamicin.

## Data Availability

The data presented in this study are available on request from the corresponding author. The data are not publicly available as they are still being used for other research works.
